# Case Report: Full recovery in severe ParvovirusB19 myocarditis with DCM phenotype: the impact of rASD and PAB

**DOI:** 10.3389/fped.2025.1579212

**Published:** 2025-06-06

**Authors:** T. Logeswaran, H. Akintürk, M. Müller, L. Rueblinger, K. Gummel, K. Klingel, C. Jux, B. Steinbrenner, D. Schranz

**Affiliations:** ^1^ Department of Pediatric Cardiology, Intensive Care Medicine and Congenital Heart Disease, Pediatric Heart Center, Justus Liebig University, Giessen, Germany; ^2^ Department of Pediatric Cardiac Surgery and Congenital Heart Disease, Pediatric Heart Center, Justus Liebig University, Giessen, Germany; ^3^ Department of Pediatric Cardiac Anesthesiology, Pediatric Heart Center, Justus Liebig University, Giessen, Germany; ^4^ Cardiopathology, Institute for Pathology and Neuropathology, University Hospital Tübingen, Tübingen, Germany; ^5^ Department of Pediatric Cardiology, Goethe University, Frankfurt, Germany

**Keywords:** B19V, myocarditis, dilated cardiomyopathy, restrictive ASD, pulmonary artery banding (PAB), case report, functional recovery

## Abstract

**Background:**

The incidence of parvovirus B19 (B19 V)-associated myocarditis progressing to dilated cardiomyopathy (DCM) is on the rise. We hypothesize that a comprehensive treatment regimen enables cardiac regeneration in young patients with life-threatening B19 V myocarditis.

**Methods:**

Four patients with clinical and imaging evidence of DCM were referred due to suspected myocarditis. An endomyocardial biopsy (EMB) confirmed the diagnosis. The diastolic dysfunction associated with heart failure and reduced left ventricular ejection fraction (HFrEF) was established invasively. Before surgical pulmonary artery banding (PAB), a transcatheter procedure was performed to create a restrictive atrial defect (rASD).

**Results:**

The drug-treated patients (ages 15–26 months) had a mean LV-EF of 22.5% (20%–25%), a left ventricular end-diastolic diameter (LVEDD) of 49 (45–51) mm (Z-score >5), and elevated LVED pressures (>18 mmHg). EMB revealed B19V-associated acute/subacute or chronic active myocarditis with characteristics of DCM. Drug therapy, including immunoglobulins and creating a rASD, resulted in clinical improvement and enhanced right ventricular function. However, LV enlargement and dysfunction persisted. Four weeks after surgical PAB, all patients showed improvement and were discharged home. The pressure gradient across the PAB ranged from 40 to 45 mmHg, and LVEDD decreased to a mean z-score of +3.5. Within three to six months, LVEDD normalized, and LV-EF increased to a mean of 63% (range: 57%–68%). Clinical and cardiac improvements were sustained over a median follow-up of 7.5 years.

**Conclusion:**

A holistic treatment approach allows functional regeneration in B19 V myocarditis with obvious end-stage DCM. Restrictive ASD creation is required before surgical PAB when HFrEF is associated with a diastolic dysfunction component.

## 1 Introduction

ParvoB19 (B19 V) myocarditis significantly impacts morbidity and mortality (1). While advanced treatment options such as ventricular assist devices (VAD) for bridging to recovery are not universally accessible (2) and are highly cost-intensive (3), existing literature supports the potential of full recovery in cases of DCM-like myocarditis (4), underscoring the importance of preserving native myocardial function. This case series highlights successful acute management and demonstrates long-term follow-up, revealing sustained normal cardiac function even after partial debanding.

Unlike enteroviruses that cause lymphocytic myocarditis by infecting cardiomyocytes, B19 V primarily targets endothelial cells within the myocardium. This often results in a compromised microcirculation with secondary myocyte damage and inflammation (5). Moreover, the infection triggers several virus-associated signaling pathways that induce an inflammatory response and apoptosis. Additionally, NS1 stimulates the production of proinflammatory cytokines such as IL6 and TNF-alpha, further increasing the inflammatory milieu within the myocardium and thereby contributing to a progression of myocardial dysfunction (6, 7).

The ongoing inflammatory process accelerates left ventricular dilation and dysfunction, contributing to various complications, including disease progression with persistent signs of congestive heart failure (8). The high prevalence of B19 V (30%–35%) in cases of dilated cardiomyopathy (DCM) suggests that DCM may develop due to previous B19V-associated myocarditis (9).

We hypothesize that a holistic treatment regimen that combines personalized drug therapy with a transcatheter-surgical approach—specifically, the creation of a restrictive ASD (rASD) followed by the placement of a reversible pulmonary artery banding (rPAB)—can lead to positive outcomes, even in patients with advanced or life-threatening P19 V myocarditis.

## 2 Case description

Between September 2013 and 2023, 14 patients with myocarditis-associated DCM were referred for heart transplantation (HTx) evaluation. Four of these patients, who had PCR-confirmed B19 V myocarditis, were reviewed retrospectively. All patients met the hospitalization inclusion criteria: aged under three years, severe left ventricle dysfunction with EF < 30%, LVEDD with a z-score ≥4, and no improvement despite heart failure therapy, including inotropic support. These patients were managed using a hybrid transcatheter-surgical approach that involved creating a restrictive atrial septal defect (ASD), followed by surgical pulmonary artery banding (PAB).

Clinical symptoms, echocardiographic, and magnetic resonance imaging (MRI) data were used to diagnose DCM. Myocarditis was diagnosed through histopathological analysis using endomyocardial biopsy (EMB), which involved PCR to detect viral DNA/RNA. Three to four biopsy samples were taken from the right interventricular septum.

The tissue samples were analyzed using histology, immunohistochemistry, and molecular pathology techniques. Acute lymphocytic myocarditis was defined by elevated numbers of CD3+ T lymphocytes (≥ 25/mm^2^) and increased numbers of CD68 + macrophages in the presence of myocyte necrosis. Ongoing myocarditis was defined as lymphocytic inflammation ≥14 CD3+ T cells and CD68 + macrophages with associated myocyte necrosis and fibrosis. Chronic myocarditis was identified based on inflammation and focal or diffuse fibrosis without myocyte necrosis, following the Dallas criteria (10, 11).

Viral genome detection using nested PCR was conducted on heart tissue and blood samples, specifically targeting enteroviruses (EV), B19 virus (B19 V), adenoviruses (ADV), human herpesviruses 6 and 7 (HHV-6/7), human cytomegalovirus (HCMV), and Epstein–Barr virus (EBV). RNA extracted from EMBs was analyzed for EV, while DNA was evaluated for the other viruses. Total nucleic acids were isolated using the QIAamp DNA Mini Kit and the QIAamp Viral RNA Mini Kit (Qiagen, Hilden, Germany), following the manufacture's protocol. Tissue samples were homogenized prior to extraction to ensure optimal yield. The effectiveness of DNA/RNA extraction was validated through the amplification of GAPDH gene sequences, and the specificity of amplification was confirmed via Sanger sequencing (5).

All patients underwent an invasive hemodynamic assessment. The hemodynamics confirmed the suspicion of a diastolic dysfunctional component in DCM patients with dominant heart failure with reduced ejection fraction (HFrEF). In patients with left ventricular end diastolic pressure ≥15 mmHg, restrictive ASD was created via transcatheter balloon atrioseptostomy using standard Brockenbrough technique followed by controlled balloon dilatation to achieve restrictive interatrial communication with a left-to-right shunting (12, 13). The goal was to reduce left atrial pressure and pulmonary venous congestion.

Cardiac MRI, including T2-weighted and T1-weighted sequences before and after intravenous gadolinium administration, was performed using a 3-Tesla Siemens MRI system (Siemens Medical System, Erlangen, Germany). It was performed before and after PAB and assessed the following parameters: LVEF, RVEF, late enhancement, and volumetry of the ventricles.

Echocardiographic assessment included LV-EF (M-mode and Simpson biplane), LVEDD with z-score, mitral valve regurgitation (graded by color Doppler), RV function by TAPSE, interventricular septal motion (including septal shift), and flow across the ASD or PFO with gradient estimation. Serial follow-up included the same parameters at discharge and during outpatient visits. Severe left ventricular dysfunction was defined as an LV-EF of less than 30% despite heart failure medication, including inotropic support. Left ventricular dilation was characterized by an LVEDD z-score greater than +2 (14) Z-score data for Pediatric Cardiology parameters (Cardio Z®) were based on Evelina Children's Hospital, London, UK.

## 3 Clinical findings and outcome

B19 V myocarditis was confirmed in all four patients. All suffered from a severe clinical course with persistent cardiac impairment. Heart failure was treated for four to six weeks, including inotropic support, before referral to our center. Based on the modified Ross Functional Class (FC) all four patients were in Class III-IV, and all patients were transferred as eligible candidates for HTx (Supplementary Table 1a+b).

Initial echocardiographic assessment, conducted while patients were on anti-congestive therapy including loop diuretics and with inotropic infusion, revealed severe LV dysfunction, with a mean LV-EF of 22.5%, increased LVEDD (mean: 49 mm; z-score >4), and moderate to severe mitral valve regurgitation, along with left atrial dilatation in all patients.

Cardiac MRI confirmed the reduced LV function, with a mean LV-EF of 24% in all patients. Right ventricular function remained within the normal range, except for patient #2, who had a reduced RV-EF of 35%. Late Gadolinium enhancement (LGE) was observed in patients #2 and #3. The initial laboratory tests showed elevated BNP serum levels (Supplementary Table 1a).

After transfer to our department, heart failure therapy was adapted. ß1-receptor-blockers were started to reduce the endogenous and exogenous impacts of catecholamines on myocardial apoptosis and oxygen demand. ACE inhibitors and spironolactone were used routinely as antifibrotic treatments in heart failure and to promote reverse remodeling (15).

Clinical improvement was observed in three patients after creating a transcatheter restrictive atrial septal defect (rASD) and in one patient after percutaneous dilatation of the patent foramen ovale (PFO). These interventions relieved the clinical symptoms of pulmonary venous congestion, thereby reducing the need for loop diuretics. Notably, RV function normalized after the development of rASD in patient #2. Despite maximal heart failure therapy, which included continuous infusion of milrinone and intermittent application of levosimendan, there was no improvement in LV function and morphology after four weeks. As a result, the decision to proceed with a surgical pulmonary artery banding was made.

Surgical Pulmonary artery banding was performed using a PTFE band, designed to decrease the pulmonary circumference by about 50%. This procedure was conducted under continuous hemodynamic and transesophageal echocardiography (TEE) monitoring. A polyethylene 21G (arterial) cannula is placed within the right ventricle through a transmural puncture and secured by a purse-string suture. The gradually tightehing of the pulmonary circumference is monitored by assessing the right ventricular pressure to systemic artery pressure (RVP/SAP) ratio, aiming of a target ratio of 0.6, while observing a leftward septal shift via TEE, ensuring that systemic arterial pressure remained uncompromised, as described by Schranz et al. in 2019 (16).

After PAB, clinical FC and echocardiographic parameters improved in all patients, allowing them to be discharged home four weeks after surgery. The continuous wave (CW) Doppler measurement of the pressure gradient across the PAB was between 40 and 50 mmHg at discharge. At follow-up three to six months later, all patients’ LV function and morphology had normalized.

Percutaneous debanding was performed after a median of 3.5 years, once LV function had normalized and RV pressure equaled or exceeded LV pressure. Under angiographic guidance, a graded balloon dilation technique was employed, with balloon diameters up to the size of the pulmonary valve annulus. Follow-up EMB was performed without complications in three patients.

At a median follow-up of 7.5 years, all patients continued to show normal LV-EF (mean: 60%; range: 55%–65%) with normalized LVEDD (mean: 34; range: 32–35 mm; z-score +0.5). The initial moderate mitral valve regurgitation had resolved in all patients. During the last follow-up, all patients were classified as being in Ross Functional Class I (Supplementary Table 2).

### 3.1 Results of endomyocardial biopsies

Endomyocardial biopsies were performed at various stages of myocardial inflammation and disease progression, starting with the first biopsy before PAB placement, followed by follow-up EMB at later intervals. The results of these biopsies provided valuable insights into each patient's evolving myocarditis pathology and progression to DCM (Supplementary Table 3).

*Patient #1 (age at diagnosis: 15 months).* Two biopsies were performed three and six weeks after the initial diagnosis. The initial biopsy confirmed acute lymphocytic myocarditis without evidence of fibrosis, suggesting a potentially reversible inflammatory proces. However the clinical trajectory was marked by persistent symptoms of heart failure and inability to wean from inotropic support (milrinone) alongside significant left ventricular dysfunction. Given the absence of clinical improvement, a repeat EMB was conducted, which revealed the development of myocardial fibrosis, a recognized marker of maladaptive remodeling, and thereby prompting the decision to proceed with a PAB.

*Patient #2 (age at diagnosis: 26 months).* Due to a late referral to our center, the first biopsy was performed eight weeks after the initial diagnosis. EMB revealed lymphocytic inflammation and extensive fibrosis but no myocyte necrosis, consistent with active chronic myocarditis. A follow-up EMB was carried out five years after PAB during partial debanding. There was no sign of lymphocytic activity at this stage, but moderate fibrosis was still present, consistent with DCM.

*Patient #3 (age at diagnosis: 17 months).* The first EMB was conducted three weeks after the clinical diagnosis and confirmed acute myocarditis with myocyte necrosis and severe lymphocytic inflammation. A follow-up EMB was performed six weeks following persistent dysfunction despite cardiac drug therapy (including inotropic support). This biopsy revealed persistent lymphocytic activity and myocyte necrosis with areas of fibrosis, suggesting an ongoing inflammatory process. Based on these findings, the decision was made to proceed with the PAB. A follow-up EMB performed five years after PAB during partial transcatheter debanding showed no signs of inflammation or necrosis, with minor fibrosis.

*Patient #4 (age at Diagnosis, 16 months).* The first EMB was conducted two weeks after admission, showing ongoing myocarditis with persistent B19 V infection, inflammation, myocyte necrosis, and extensive fibrosis. A follow-up biopsy, performed one year after PAB during partial transcatheter debanding, revealed chronic myocarditis without necrosis and only minor fibrosis at this stage.

## 4 Discussion

The case series of severely ill patients with B19 V myocarditis progressing to DCM highlights the potential for functional cardiac recovery in younger patients. Our data emphasize the importance of a personalized, holistic treatment approach that considers the patient's age and hemodynamics, which can significantly affect the heart's regenerative potential. Our unique pre- and post-PAB histological findings reinforce previous observations of cardiac recovery, even in end-stage DCM cases. This approach may provide an opportunity to minimize the need for assistive ventricular devices.

### 4.1 Parvovirus B19 myocarditis and its role in pediatric DCM

B19 V has been increasingly recognized as a potential cause of myocarditis (17). In pediatric patients, myocarditis is often associated with better ventricular remodeling and survival outcomes than idiopathic DCM (17). However, severe LV dysfunction and LVEDD z-scores greater than +2 are linked to a reduced likelihood of full recovery, as indicated by echocardiographic normalization (17). Chronic inflammation exacerbates cardiac remodeling, which can perpetuate further functional decline and worsen heart failure (10).

These findings highlight the urgent need to identify the type of myocarditis and possible underlying virus infections. Differentiating these conditions has significant implications for prognosis and therapeutic strategies. Myocardial fibrosis reflects a shift from acute inflammation toward chronic structural remodeling, which is strongly associated with progressive ventricular dysfunction and adverse outcomes (18). The presence of fibrosis, particularly in the setting of ongoing left ventricular dilatation (19), indicates a transition toward irreversible myocardial damage if not promptly addressed. Based on these findings, the decision was made to proceed with pulmonary artery banding (PAB) to induce left ventricular reconditioning, support reverse remodeling, and potentially halt or slow the fibrotic process.

Recent data suggest that patients with severe acute myocarditis may face a greater urgency for HTx listing. Between 2009 and 2019, over 50% of children under five were listed for HTx, and more than 38% were under one year old.

However, despite some patients recovering, nearly two-thirds of those under one year old remain on the transplant list after six months (17), highlighting the ongoing challenges in managing this patient population. Furthermore, there is an increased risk of early graft loss and poorer long-term survival in HTx recipients with myocarditis compared to those with DCM (17). These findings emphasize that persistent B19 V infection can lead to inadequate resolution with persistent inflammation. This is further supported by the observed effects of B19 V (20) and other viral infections, such as SARS-CoV-2 (21), on the cardiovascular system, which may contribute to poor outcomes in transplant recipients due to heightened immune activation and graft failure.

Although the small sample size (*n* = 4) limits the generalizability of these findings, these observations raise important questions and indicate areas for further research.

### 4.2 Key observations and hypotheses

#### 4.2.1 Reversibility of severe B19 V myocarditis leading to DCM, impact of PAB

Severe B19 V myocarditis that progresses to DCM may be reversible as long as an individualized therapeutic approach is implemented. For young patients with LV-DCM, an essential criterion for surgical PAB is preserved RV function. Creating restrictive atrial communication before PAB can help manage complications arising from additional diastolic dysfunction, pulmonary hypertension, and right ventricular dysfunction (13, 14).

In young patients with advanced DCM, PAB induces RV pressure loading, improving ventricular interaction (VVI), re-synchronizing LV contraction, and enhancing preload effects on the failing LV. This ultimately improves overall cardiac architecture and function (22). Importantly, PAB should be paired with a tailored cardioprotective drug therapy, particularly the use of selective β1-adrenoreceptor blockers (13).

#### 4.2.2 Role of invasive endomyocardial biopsy

This case series highlights the crucial role of invasive EBM in diagnosing and evaluating B19 V myocarditis and monitoring disease progression. The biopsies reveal various histopathological patterns associated with B19 V myocarditis, all presenting a similar LV-DCM phenotype. Follow-up biopsies provided vital insights into the recovery process. The findings indicate that the activation of fibrosis-promoting pathways begins at a young age and can intensify beyond infancy (23), underscoring the significance of early interventions to slow fibrotic progression.

#### 4.2.3 EBM as a tool for risk stratification and monitoring

EBM performed before PAB and during follow-up allowed for a better understanding of the initial inflammation and fibrosis characteristics. Ongoing inflammation, marked by lymphocytic activity and fibrosis, has been linked to negative remodeling and worsening LV dilation and dysfunction (8). Myocardial fibrosis is characterized by an excessive accumulation of collagen in the myocardium. There are two forms of myocardial fibrosis: replacement fibrosis and interstitial fibrosis. Importantly, both types of fibrosis can coexist and are independent predictors of adverse cardiac outcomes if left untreated (18). Therefore, EBM can serve as a valuable tool in risk stratification and ongoing monitoring of these patients.

### 4.3 Conclusion

Our findings support the positive impact of PAB in promoting functional recovery of the dilated left myocardium, particularly in young patients with life-threatening B19 V myocarditis, leading to DCM (12). This is the first report demonstrating PAB-related LV recovery, confirmed by further EBM-based histopathological examinations, even during mid-term follow-up. Our clinical data shows functional regeneration and cardiac recovery following severe myocardial damage in infants and young children aged between one and three years (16, 24, 25). These results are consistent with theoretical models and animal studies demonstrating similar reverse remodeling processes (22, 26).

Although the molecular mechanisms underlying the interventricular crosstalk after PAB placement remain unclear, our histopathological data suggest that the VVI mechanism corresponds well with the observed clinical and imaging outcomes. Future research is needed to better understand the molecular pathways involved in this recovery process and to determine whether PAB, when combined with specific medications, could serve as a prophylactic or therapeutic option in other cases of severe myocarditis and DCM (19).

## Data Availability

The original contributions presented in the study are included in the article/Supplementary Material, further inquiries can be directed to the corresponding author.
