# Supplementary material for: Case Report: Full recovery in severe ParvovirusB19 myocarditis with DCM phenotype: the impact of rASD and PAB
Source: Front Pediatr. 2025 Jun 6;13:1579212. doi: 10.3389/fped.2025.1579212 (PMC12179178; doi:10.3389/fped.2025.1579212)
Supplement: Supplementary file 1 [file Presentation1.pptx]

## Slide 1
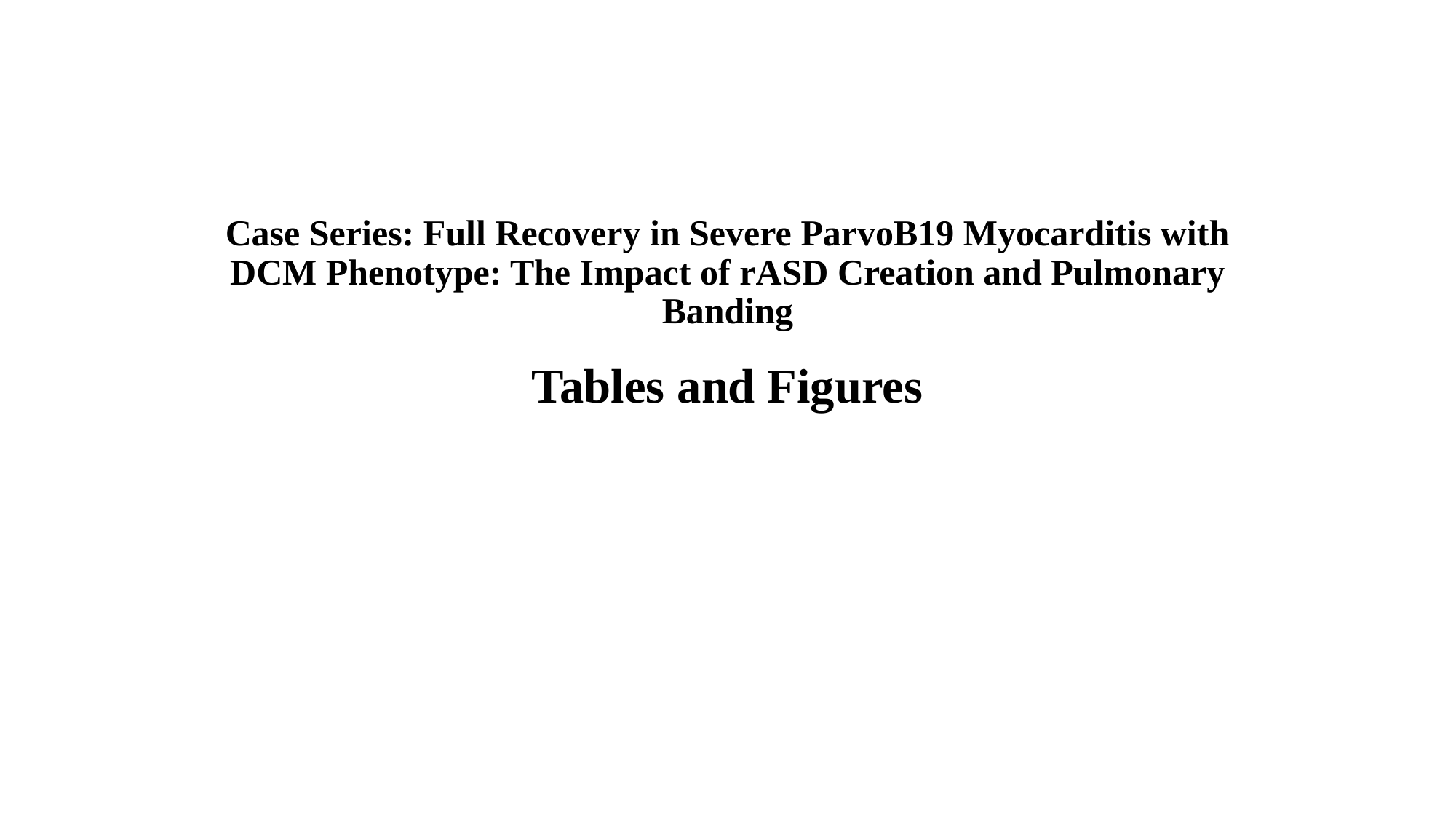

# Case Series: Full Recovery in Severe ParvoB19 Myocarditis with DCM Phenotype: The Impact of rASD Creation and Pulmonary Banding Tables and Figures

## Slide 2
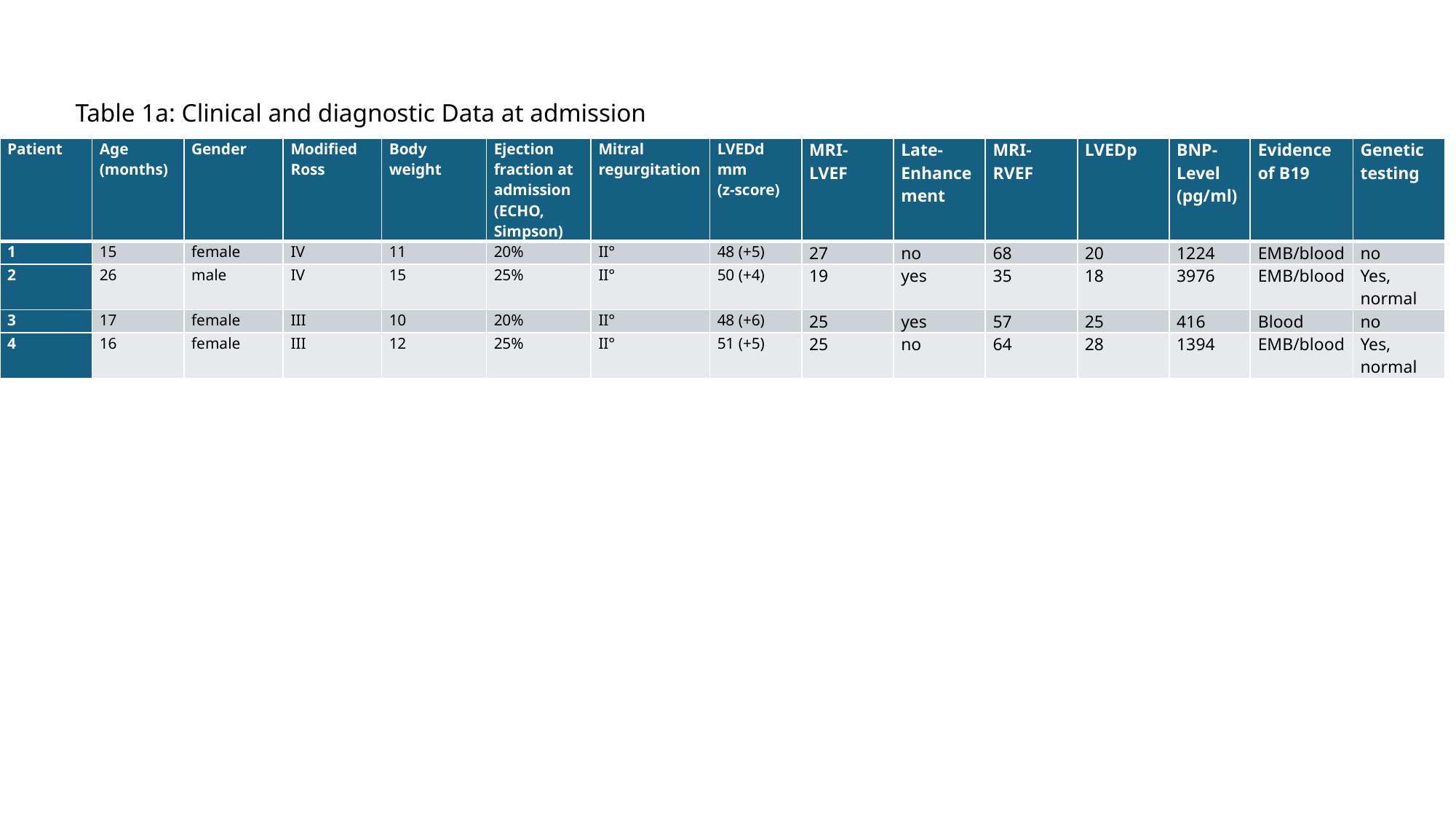

Table 1a: Clinical and diagnostic Data at admission
| Patient | Age (months) | Gender | Modified Ross | Body weight | Ejection fraction at admission (ECHO, Simpson) | Mitral regurgitation | LVEDd mm (z-score) | MRI-LVEF | Late-Enhancement | MRI-RVEF | LVEDp | BNP-Level (pg/ml) | Evidence of B19 | Genetic testing |
| --- | --- | --- | --- | --- | --- | --- | --- | --- | --- | --- | --- | --- | --- | --- |
| 1 | 15 | female | IV | 11 | 20% | II° | 48 (+5) | 27 | no | 68 | 20 | 1224 | EMB/blood | no |
| 2 | 26 | male | IV | 15 | 25% | II° | 50 (+4) | 19 | yes | 35 | 18 | 3976 | EMB/blood | Yes, normal |
| 3 | 17 | female | III | 10 | 20% | II° | 48 (+6) | 25 | yes | 57 | 25 | 416 | Blood | no |
| 4 | 16 | female | III | 12 | 25% | II° | 51 (+5) | 25 | no | 64 | 28 | 1394 | EMB/blood | Yes, normal |

## Slide 3
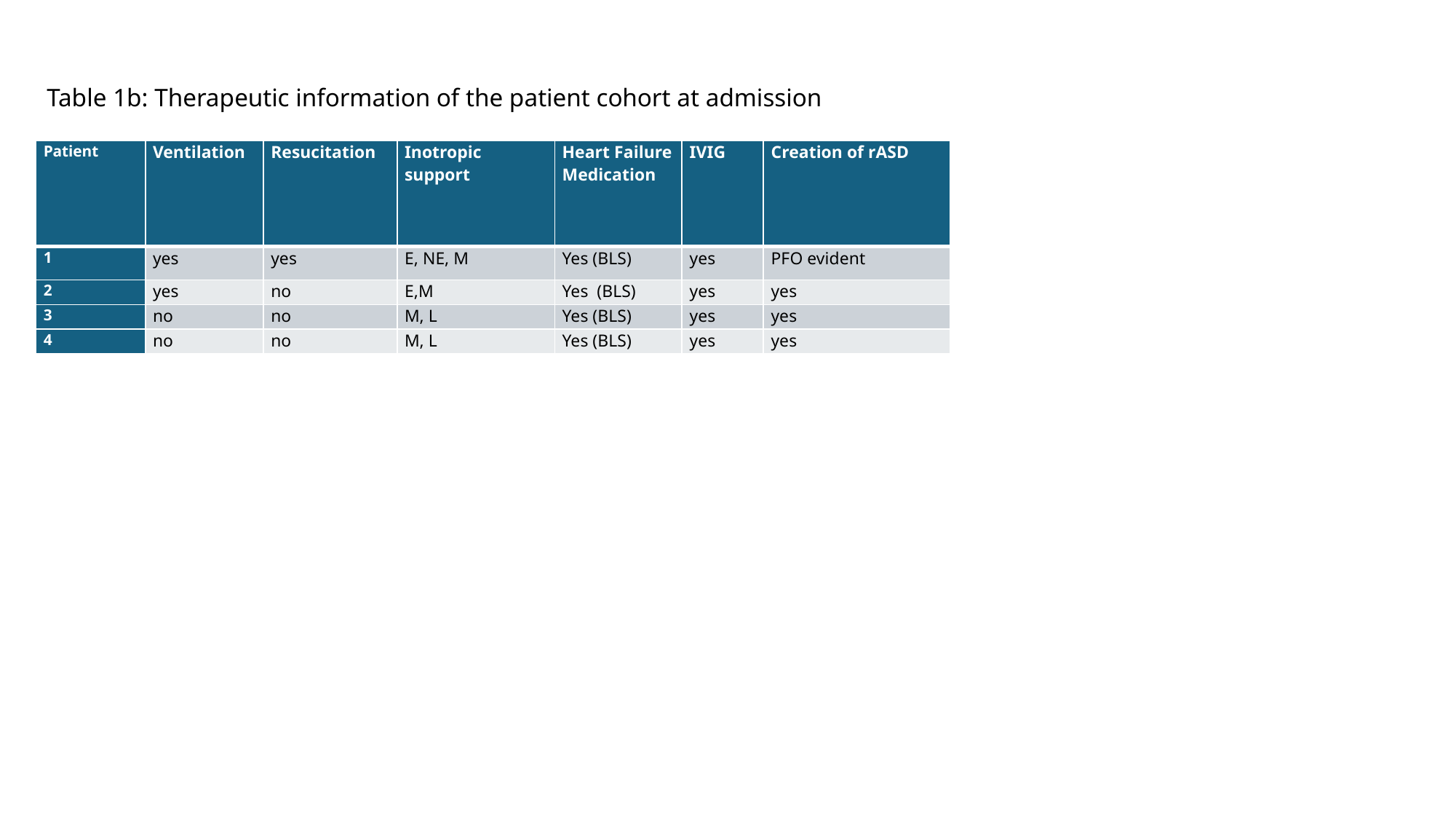

Table 1b: Therapeutic information of the patient cohort at admission
| Patient | Ventilation | Resucitation | Inotropic support | Heart Failure Medication | IVIG | Creation of rASD |
| --- | --- | --- | --- | --- | --- | --- |
| 1 | yes | yes | E, NE, M | Yes (BLS) | yes | PFO evident |
| 2 | yes | no | E,M | Yes (BLS) | yes | yes |
| 3 | no | no | M, L | Yes (BLS) | yes | yes |
| 4 | no | no | M, L | Yes (BLS) | yes | yes |

## Slide 4
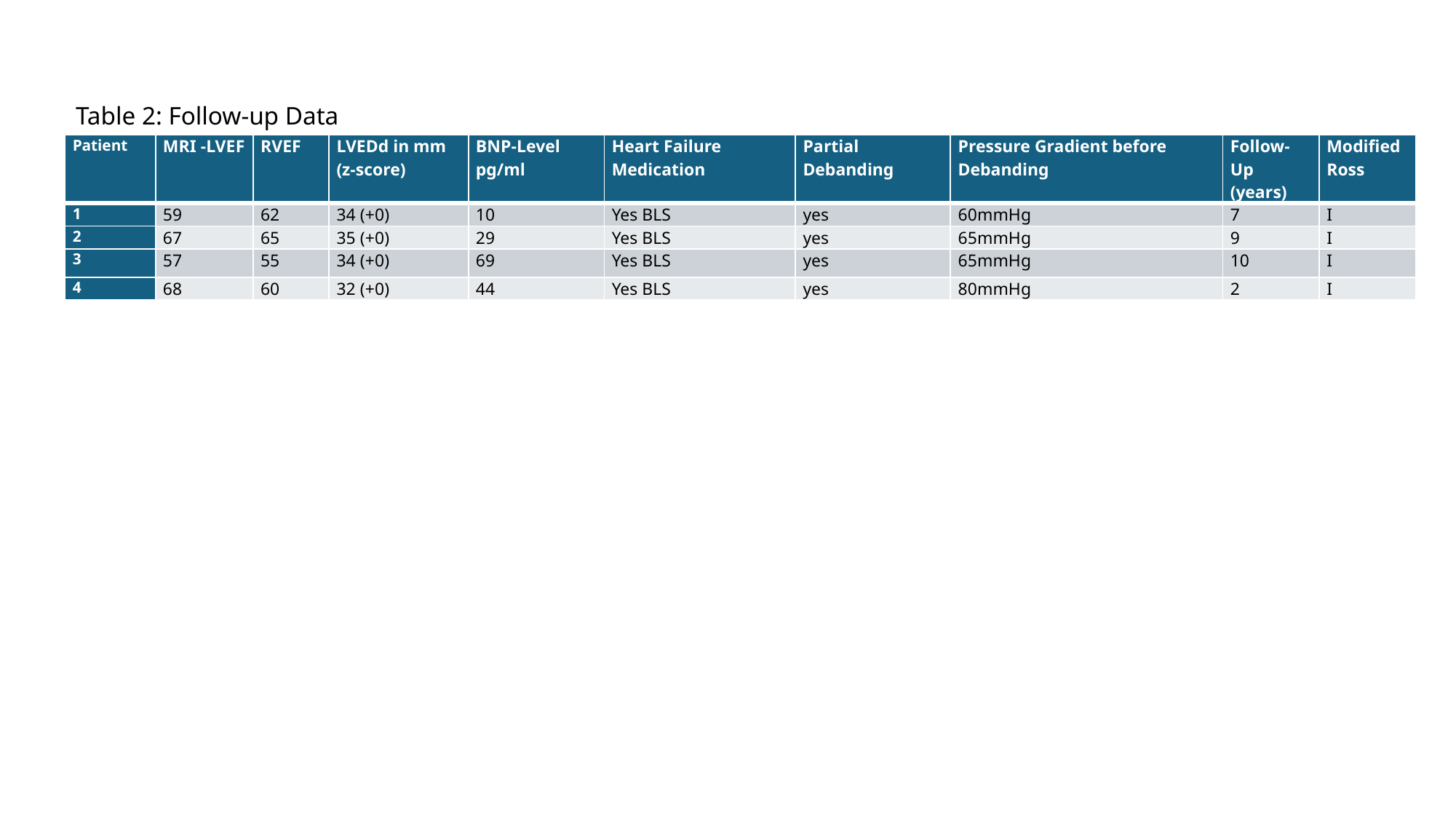

Table 2: Follow-up Data
| Patient | MRI -LVEF | RVEF | LVEDd in mm (z-score) | BNP-Level pg/ml | Heart Failure Medication | Partial Debanding | Pressure Gradient before Debanding | Follow-Up (years) | Modified Ross |
| --- | --- | --- | --- | --- | --- | --- | --- | --- | --- |
| 1 | 59 | 62 | 34 (+0) | 10 | Yes BLS | yes | 60mmHg | 7 | I |
| 2 | 67 | 65 | 35 (+0) | 29 | Yes BLS | yes | 65mmHg | 9 | I |
| 3 | 57 | 55 | 34 (+0) | 69 | Yes BLS | yes | 65mmHg | 10 | I |
| 4 | 68 | 60 | 32 (+0) | 44 | Yes BLS | yes | 80mmHg | 2 | I |

## Slide 5
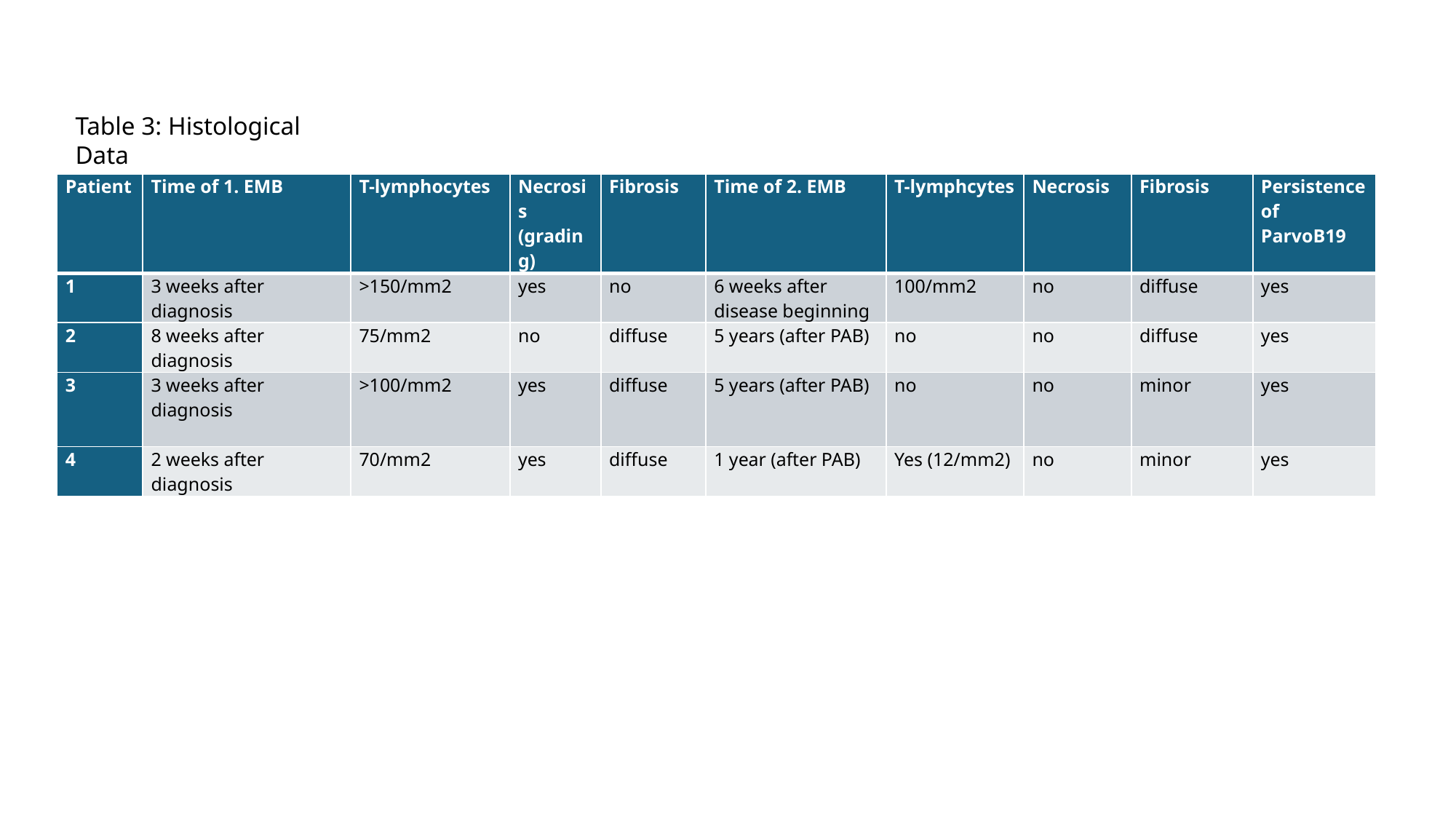

Table 3: Histological Data
| Patient | Time of 1. EMB | T-lymphocytes | Necrosis (grading) | Fibrosis | Time of 2. EMB | T-lymphcytes | Necrosis | Fibrosis | Persistence of ParvoB19 |
| --- | --- | --- | --- | --- | --- | --- | --- | --- | --- |
| 1 | 3 weeks after diagnosis | >150/mm2 | yes | no | 6 weeks after disease beginning | 100/mm2 | no | diffuse | yes |
| 2 | 8 weeks after diagnosis | 75/mm2 | no | diffuse | 5 years (after PAB) | no | no | diffuse | yes |
| 3 | 3 weeks after diagnosis | >100/mm2 | yes | diffuse | 5 years (after PAB) | no | no | minor | yes |
| 4 | 2 weeks after diagnosis | 70/mm2 | yes | diffuse | 1 year (after PAB) | Yes (12/mm2) | no | minor | yes |

## Slide 6
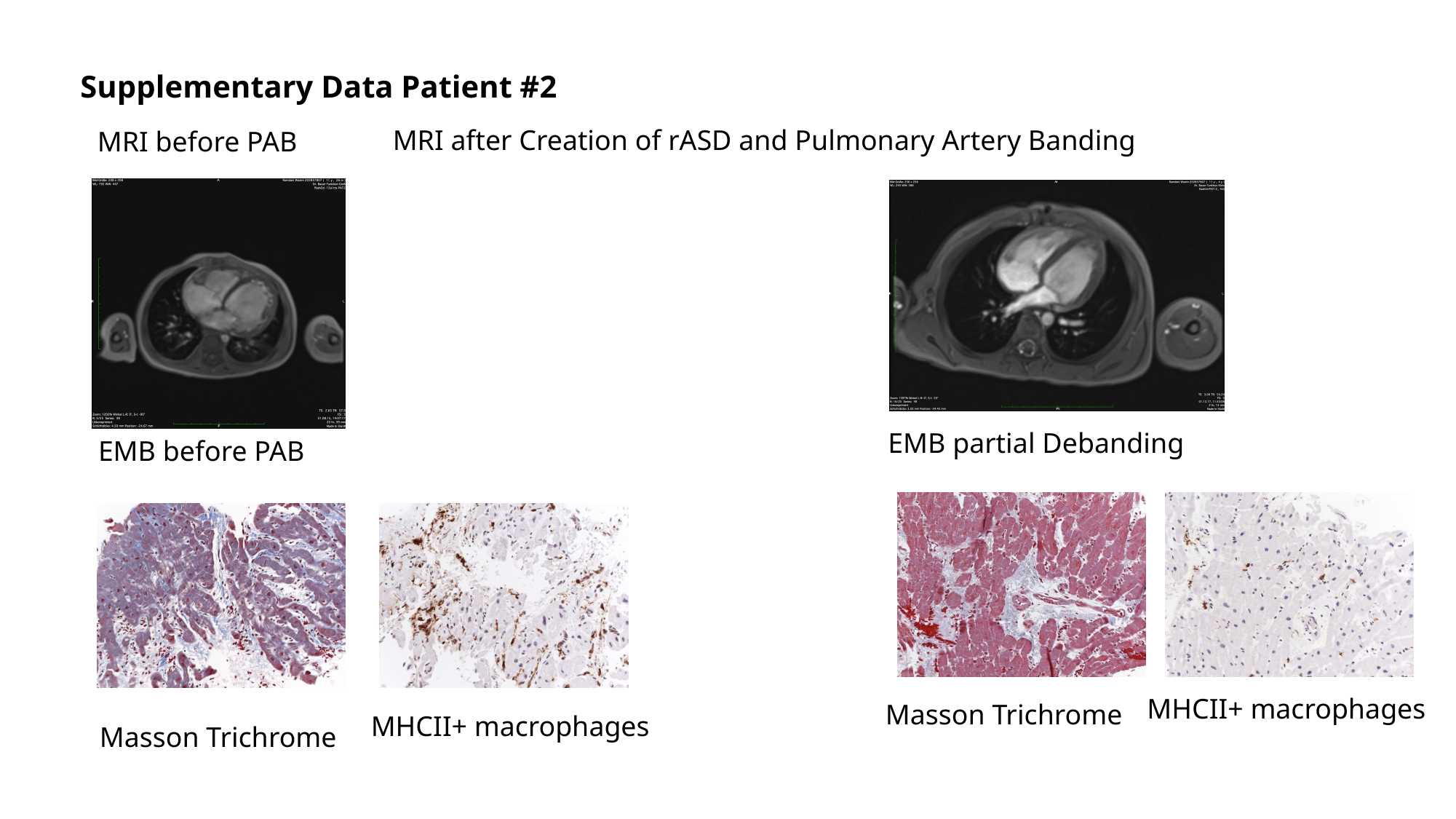

Supplementary Data Patient #2
MRI after Creation of rASD and Pulmonary Artery Banding
MRI before PAB
EMB partial Debanding
EMB before PAB
MHCII+ macrophages
Masson Trichrome
MHCII+ macrophages
Masson Trichrome
